# Oxidative Stress Increases Endogenous Complement-Dependent Inflammatory and Angiogenic Responses in Retinal Pigment Epithelial Cells Independently of Exogenous Complement Sources

**DOI:** 10.3390/antiox8110548

**Published:** 2019-11-13

**Authors:** Timon-Orest Trakkides, Nicole Schäfer, Maria Reichenthaler, Konstanze Kühn, Ricardo J. M. G. E. Brandwijk, Erik J. M. Toonen, Florian Urban, Joachim Wegener, Volker Enzmann, Diana Pauly

**Affiliations:** 1 Experimental Ophthalmology, Eye clinic, University Hospital Regensburg, 93053 Regensburg, Germany; timon-orest.trakkides@stud.uni-regensburg.de (T.-O.T.); nicole.schaefer@ukr.de (N.S.); maria.reichenthaler@googlemail.com (M.R.); konstanze_kuehn@t-online.de (K.K.); 2 R&D Department, Hycult Biotech, 5405 PD Uden, The Netherlands; r.brandwijk@hycultbiotech.com (R.J.M.G.E.B.); e.toonen@hycultbiotech.com (E.J.M.T.); 3 Institute of Analytical Chemistry, Chemo- and Biosensors, University of Regensburg, 93053 Regensburg, Germany; florian.urban@ur.de (F.U.); joachim.wegener@ur.de (J.W.); 4 Department of Ophthalmology, University Hospital of Bern and Department of Biomedical Research, University of Bern, 3010 Bern, Switzerland; volker.enzmann@insel.ch

**Keywords:** oxidative stress, retinal pigment epithelial cells, complement system, inflammasome, foxp3, olaparib

## Abstract

Oxidative stress-induced damage of the retinal pigment epithelium (RPE) and chronic inflammation have been suggested as major contributors to a range of retinal diseases. Here, we examined the effects of oxidative stress on endogenous complement components and proinflammatory and angiogenic responses in RPE cells. ARPE-19 cells exposed for 1–48 h to H_2_O_2_ had reduced cell–cell contact and increased markers for epithelial–mesenchymal transition but showed insignificant cell death. Stressed ARPE-19 cells increased the expression of complement receptors CR3 (subunit CD11b) and C5aR1. CD11b was colocalized with cell-derived complement protein C3, which was present in its activated form in ARPE-19 cells. C3, as well as its regulators complement factor H (CFH) and properdin, accumulated in the ARPE-19 cells after oxidative stress independently of external complement sources. This cell-associated complement accumulation was accompanied by increased *nlrp3* and *foxp3* expression and the subsequently enhanced secretion of proinflammatory and proangiogenic factors. The complement-associated ARPE-19 reaction to oxidative stress, which was independent of exogenous complement sources, was further augmented by the poly(ADP-ribose) polymerase (PARP) inhibitor olaparib. Our results indicate that ARPE-19 cell-derived complement proteins and receptors are involved in ARPE-19 cell homeostasis following oxidative stress and should be considered as targets for treatment development for retinal degeneration.

## 1. Introduction

One of the most oxidative environments in the body is the retinal pigment epithelium (RPE) [1], which is in close contact with the photoreceptors and maintains visual function [2]. Low levels of reactive oxygen species are required to maintain physiological functions [3], but the combination of visible light exposure, elevated metabolic activity, the accumulation of oxidized lipoproteins, and decreased antioxidant functions during aging make the retinal tissue vulnerable to oxidative stress [4,5]. Oxidative damage to the RPE has therefore been identified as a contributing factor to different retinal degenerative diseases, such as age-related macular degeneration or Stargardt disease [6,7,8,9,10,11].

Consistent with these observations, constant oxidative stress can trigger chronic inflammation, subsequently leading to cellular damage in the RPE/retina [6,12]. On the basis of genetic polymorphisms in genes of the complement system, systemic complement activation, and local complement deposition in degenerative retinal tissue, a contribution of the complement system to oxidative stress-related retinal degeneration has been hypothesized [7,13,14]. The complement system is composed of over 40 proteins, which bridge the innate and adaptive immune defense [15]. The main functions are (I) the removal of damaged cells, (II) protection against invading pathogens, and (III) the attraction of immune cells.

Besides the traditional view, evidence is accumulating that complement components are also involved in physiological processes, such as responses to oxidative stress and cellular maintenance [6]. The complement system comprises several soluble and membrane-bound proteins and receptors, which can be produced by a number of cells (including nonimmune cells and extrahepatic tissue) and contribute to autocrine cell physiology [16]. The role of endogenous complement-dependent regulation of cellular homeostasis has recently been extensively studied [17]. T-cells, B-cells, and human airway epithelial cells contain intracellular stores of C3, which is endogenously cleaved into its active forms, C3a and C3b, by intracellular proteases [18,19,20]. Activated C3 is correlated with the activation of the NLR family pyrin domain containing 3 (NLRP3) inflammasome in T-effector cells [18], which leads to proinflammatory cytokine release. An antagonizing complement modulation has been described for regulatory T-cells, where C3aR and C5aR1 activation resulted in the activation of the forkhead box P3 (FOXP3) transcription factor [18,21]. The FOXP3 transcription factor acts in multimodal fashion and stimulates the release of anti-inflammatory cytokines and proangiogenic factors [21,22,23].

Oxidative stress and inflammasome activation have previously been correlated with external complement activity in RPE cells [6,24]. FOXP3 activation in RPE cells also depends on extracellularly added complement components [25]. However, an RPE-derived complement has not been discussed as a source for NLRP3 or FOXP3 modulation. Complement components can be produced by RPE cells [26], and their expression is changed under oxidative stress [27,28,29,30,31]. Further activated forms of C3 (C3a), independently of extracellular complement sources, are also secreted by RPE cells, suggesting a similar function of the complement system in RPE cells compared to T-cells [32,33,34,35].

In this study, we report that H_2_O_2_ stimulated the accumulation of complement proteins C3, complement factor H (CFH), and properdin in RPE cells and increased the expression of complement receptors C5aR1 and CR3 (subunit CD11b). This was accompanied by increased *nlrp3* inflammasome expression and the FOXP3-associated release of proangiogenic factors. Our results indicate a cell homeostatic function of cell-derived complement components that is independent of external complement receptor ligands.

## 2. Materials and Methods

### 2.1. Cell Culture and Treatment

Human male adult retinal pigment epithelium cells (ARPE-19 cells, passage 39; American Type Culture Collection, #CRL-2302) were cultivated for 6 days in cell culture flasks with Dulbecco’s modified eagle medium (DMEM/F12; Sigma-Aldrich, Darmstadt, Germany), 10% fetal calf serum (FCS; PanBiotech, Aidenbach, Germany), and 1% penicillin/streptomycin (37 °C, 5% CO_2_). Cells were trypsinized (0.05% trypsin/0.02% ethylenediaminetetraacetic acid (EDTA)) and seeded in a concentration of 1.6 × 10^5^ cells/cm^2^ (passage 39) on mouse laminin-coated (5 µg/cm^2^, Sigma-Aldrich, Darmstadt, Germany) 0.4-μm-pore polyester membrane inserts (Corning, Corning, NY, USA). Cells were cultivated for 4 weeks with apical and basal media exchanges (first-day medium with 10% FCS), remaining time medium with 5% FCS). Before treatment, the FCS concentration was reduced to 0% within 3 days (5%–2.5%–1.25%). ARPE-19 cells were treated with either 0.5 mM H_2_O_2_ for 1, 4, 24, and 48 h or with 0.5 mM H_2_O_2_ and 0.01 mM olaparib (Biomol, Hamburg, Germany) for 4 h.

### 2.2. Immunohistochemistry and Terminal Deoxynucleotidyl Transferase dUTP Nick End Labeling (TUNEL) Assay

Phosphate buffered saline (PBS)-washed, paraformaldehyde-fixated (4%, 20 min; Merck, Darmstadt, Germany) ARPE-19 cells were permeabilized (PBS/0.2% Tween20 (PBS-T), 45 min), and unspecific bindings were blocked (3% bovine serum albumin (BSA) (Carl Roth, Karlsruhe, Germany)/PBS-T, 1 h). Antigens were detected using a primary antibody (Supplementary Materials, Table S1, overnight, 3% BSA/PBS-T) and a fluorescence-conjugated antispecies antibody (Supplementary Materials, Table S1, 45 min, 3% BSA/PBS). The fluorochrome HOECHST 33342 (1:1000) was used to stain DNA. Cells were covered with fluorescence mounting medium (Dako, Agilent Technologies, Santa Clara, CA, USA). Images were taken with a confocal microscope (Zeiss, Jena, Germany).

The TUNEL assay was performed with a DeadEnd™ Fluorometric TUNEL System (Promega, Madison, WI, USA) on paraformaldehyde-fixated, washed, and permeabilized (0.2% Triton X-100 in PBS) cells. Images were taken with a confocal microscope (Zeiss, Jena, Germany).

### 2.3. Transepithelial Resistance (TER) and Cellular Capacitance

TER and cell layer capacitance were recorded online using the established cellZscope device (nanoAnalytics, Münster, Germany), as previously described [36]. The dielectric properties of empty filter inserts were determined independently and were included in the equivalent circuit used for analysis. Fitting the parameters of the equivalent circuit to the experimental data was achieved via nonlinear least-squares optimization according to the Levenberg–Marquardt algorithm.

### 2.4. Real-Time, Quantitative Polymerase Chain Reaction (RT-qPCR)

Here, mRNA was isolated using a NucleoSpin^®^ RNA/Protein kit (Macherey-Nagel, Düren, Germany). Purified mRNA was transcribed into cDNA with a QuantiTect^®^Reverse Transcription Kit (Qiagen, Hilden, Germany). Transcripts of complement components, receptors, and inflammation-associated markers were analyzed using a Rotor-Gene SYBR^®^Green PCR Kit either with QuantiTect Primer Assays (Supplementary Materials, Table S2) or in-house-designed primer pairs (Metabion, Planegg, Germany) (described in the Supplementary Materials, Table S3) in a Rotor Gene Q 2plex cycler (Qiagen, Hilden, Germany). Data were analyzed using the delta delta Ct (ddCt) method. Values were depicted on a linear scale using log-transformed scores to equally visualize increases and decreases in expression levels.

### 2.5. Western Blot

Proteins were dissolved in RIPA buffer (Sigma-Aldrich, Darmstadt, Germany) with protease and phosphatase inhibitors (1:100, Sigma-Aldrich, Darmstadt, Germany). Samples were diluted in reducing Laemmli sample buffer and denatured (95 °C, 10 min). Following sample separation in a 12% SDS-PAGE, proteins were transferred onto an activated polyvinylidene difluoride membrane using a wet-blotting system. Membranes were blocked (1 h, 5% BSA/PBS-T) and incubated with the primary antibody (Supplementary Materials, Table S1, overnight, 5% BSA/PBS-T). Peroxidase-conjugated antispecies antibodies were used for detection (Supplementary Materials, Table S1, 1 h, PBS-T). WesternSure PREMIUM Chemiluminescent Substrate (LI-COR biosciences, Lincoln, NE, USA) visualized the antigen in a Fluor Chem FC2 Imaging System (Alpha Innotech, San Leandro, CA, USA).

### 2.6. Protein Secretion Assays

Properdin levels in cell culture supernatants were determined using a sandwich ELISA, as previously described [37]. C3 quantification was performed using a newly developed C3 ELISA (cat# HK366-01; Hycult Biotech, Uden, Netherlands) according to the manufacturer’s protocol. CFH was quantified in an in-house ELISA with mouse anti-CFH monoclonal antibody (BioRad, Feldkirchen, Germany) as a capture antibody and goat anti-CFH polyclonal antibody (Merck, Darmstadt, Germany) as a detection antibody (Supplementary Materials, Table S1). A comparison of properdin, C3, and CFH levels in supernatants of lower and higher ARPE-19 passages was performed using a MILLIPLEX MAP Human Complement Panel (Merck, Darmstadt, Germany). Interleukin (IL)-1β und IL-6 concentrations were determined according to the protocol of a custom ProcartaPlex^®^ multiplex immunoassay kit (ThermoFisher, Waltham, MA, USA). IL-8 was analyzed using xMAP technology, anti-IL-8 beads (cat# 171-BK31MR2; BioRad, Feldkirchen, Germany), and anti-IL-8 biotin (cat# 171-BK31MR2; BioRad, Feldkirchen, Germany) according to the manufacturer’s protocol. The readout of the multiplex assays (IL-1β, IL-6, and IL-8) was performed using a Magpix instrument (Luminex, Austin, TX, USA). Vascular endothelial growth factor (VEGF)-α concentrations were determined using a human VEGF Quantikine ELISA Kit (R&D system, Minneapolis, MN, USA).

### 2.7. Statistics

Expression statistical analyses for the mRNA were performed using a nonparametric, unpaired Kruskal–Wallis test with Dunn’s multiple comparison correction, and protein secretion was statistically analyzed using an ordinary two-way analysis of variance ANOVA with Bonferroni’s multiple comparisons test (GraphPad Prism 7; GraphPad Software Inc., San Diego, CA, USA).

## 3. Results

### 3.1. Stressed, In Vivo-Like Cultivation of ARPE-19 Cells

**Figure 1:**
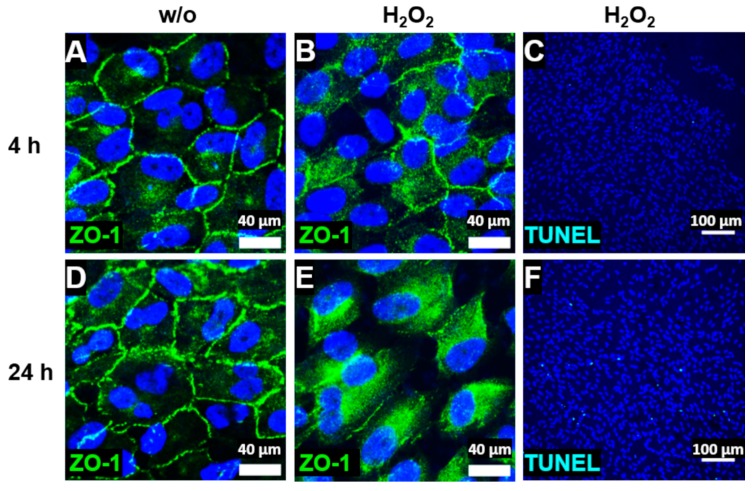
ARPE-19 cells reduced tight junctions and circumvented apoptosis under oxidative stress. (**A**,**D**) ARPE-19 cells untreated (without (w/o)) and stressed with H_2_O_2_ for (**B**,**C**) 4 h or (**E**,**F**) 24 h translocated the zonula occludens protein 1 (ZO-1, green) time-dependently from the (**A**,**D**) cell membrane to the (**B**,**E**) cytoplasm. (**C**,**F**) ARPE-19 cells treated with oxidative stress showed a minimal TUNEL-positive (light blue) apoptotic reaction after (**F**) 24 h.

We investigated cellular stress response and cell-specific complement expression in a cell line of human RPE cells, the ARPE-19 cell line. Aged ARPE-19 cells of passage 39 were cultivated under in vivo-like, unstressed conditions. This was visualized by staining zonula occludens 1 (ZO-1), an important protein for cell–cell contact, and this showed the formation of stable tight junctions and mainly mononuclear, polarized cell growth on transwell filters (Figure 1A,D). Stable transepithelial resistance (TER), a measure of the cell layer’s barrier function, and the cell layer’s capacitance, which is indicative of the expression of membrane protrusions such as microvilli and other membrane folding, were characteristics of the in vivo-like cultivated ARPE-19 cells (Supplementary Materials, Figure S1A,B). H_2_O_2_ treatment resulted in cellular stress, which was indicated by reduced cell–cell contact after 4 h (Figure 1B) and a time-dependent translocation of ZO-1 from the cell membrane to the cytoplasm after 24 h (Figure 1E). Evidence of induced cellular stress by H_2_O_2_ was also observed in the increased mRNA expression of vimentin (*vim*) and α-smooth muscle actin (*α-sma*), a typical mesenchymal marker indicating an epithelial–mesenchymal transition (Supplementary Materials, Figure S1C,D) [38,39,40]. However, the majority of the ARPE-19 cells did not undergo apoptosis under these nonlethal oxidative stress conditions, as shown by a low number of TUNEL-positive cells (Figure 1C,F), and H_2_O_2_-treated cells maintained the cell layer’s barrier function as well as the cell layer’s capacitance (Supplementary Materials, Figure S1E,F).

### 3.2. ARPE-19 Cells Increased Complement Receptor Expression under Oxidative Stress

**Figure 2:**
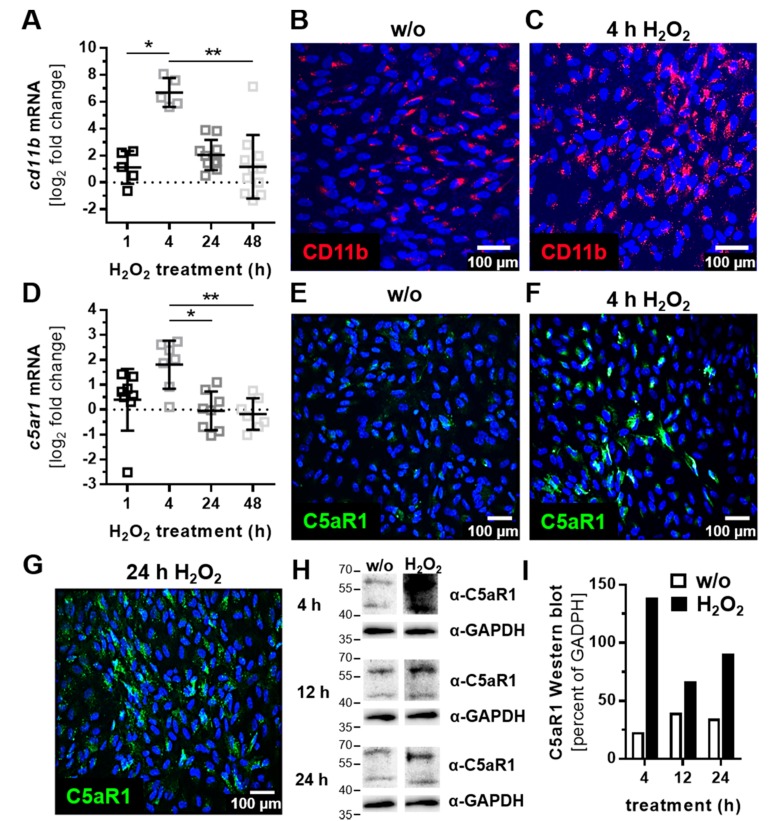
Oxidative stress increased the expression of complement receptor subunit CD11b and C5aR1 in ARPE-19 cells. (**A**) *Cd11b* mRNA expression was increased 4 h after H_2_O_2_ treatment. This effect was confirmed on a protein level by immunohistochemistry using (**B**,**C**) anti-CD11b (red) antibodies. (**D**) *C5ar1* mRNA also increased on (**D**) mRNA and (**E**–**G**) protein level (anti-C5aR1, green) in H_2_O_2_ treated cells. (**H**) Western blots of ARPE-19 cell lysates detected C5aR1 between 40 and 60 kDa after 4–24 h H_2_O_2_ treatment (full immunoblots are shown in the Supplementary Materials, Figure S3A,B; *n* = 1) (**I**) Quantitatively, C5aR1 expression was increased in H_2_O_2-_treated cells in the Western blots. (**A**,**D**) Mean with standard deviation is shown, * *p* ≤ 0.05, ** *p* ≤ 0.01. The dotted line depicts the untreated control; (**B**,**E**,**H**,**I**) w/o untreated control.

ARPE-19 cells express cellular receptors, sense the cellular environment, and can react to complement activation products. Complement receptor 3 (CR3) is a heterodimer integrin consisting of two noncovalently linked subunits (CD11b and CD18) on leukocytes/microglia, and it is activated by C3 cleavage products (iC3b, C3d, and C3dg). We detected CD11b with low expression in mRNA and low protein levels in ARPE-19 cells (Figure 2A,B). Oxidative stress increased *cd11b* mRNA expression after 4 h, which was also shown in protein levels with immunostaining (Figure 2A,C).

The activation of complement protein C5 was detected by complement receptor C5aR1, which was expressed by ARPE-19 cells (Figure 2D). H_2_O_2_ treatment increased *c5ar1* expression comparably to *cd11b* expression (Figure 2D‒F). C5aR1 protein accumulation was observed after 4 h in the cell nuclei (Figure 2F), which was more distributed in/on the cell after 24 h (Figure 2G). Increased C5aR1 protein levels were also confirmed in Western blots (Figure 2H,I).

The transcription levels of complement receptor *c3aR* were not significantly changed in H_2_O_2_-treated ARPE-19 cells (Supplementary Materials, Figure S2A).

### 3.3. Complement Proteins Accumulated in ARPE-19 Cells under Oxidative Stress

**Figure 3:**
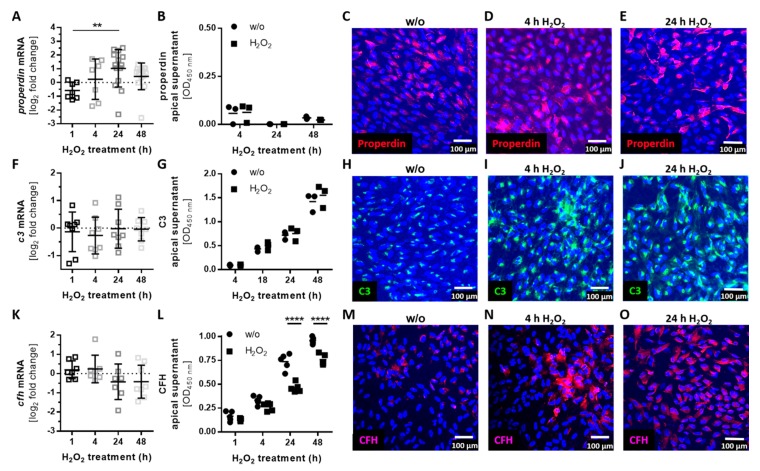
Oxidative stress induced complement component accumulation in ARPE-19 cells. (**A**) *Properdin* mRNA levels were increased 24 h following H_2_O_2_ treatment. This did not affect (**B**) apical properdin secretion, but was confirmed in the protein level by immunohistochemistry using an (**C**–**E**) anti-properdin (red) antibody. (**F**) *C3* mRNA and (**G**) apical C3 protein secretion were not altered in stressed ARPE-19 cells. Immunohistochemistry using (**H**–**J**) anti-C3 (green) antibodies showed an increase of cell-associated (**I**,**J**) C3 after oxidative stress treatment. (**K**) *Cfh* mRNA and (**L**) CFH apical protein concentration were decreased following H_2_O_2_ treatment. (**M**–**O**) Immunohistochemistry using anti-complement factor H (CFH, purple) antibodies showed an increase in cell-associated (**N**,**O**) CFH after oxidative stress treatment. Mean with standard deviation is shown, ** *p* ≤ 0.01, **** *p* ≤ 0.0001; dotted line depicts untreated control (**A**,**F**,**K**); w/o untreated control (**G**,**G**,**L**); ELISA control standard curves and protein concentrations in the basal supernatants are shown in the Supplementary Materials, Figure S4D–I.

Complement proteins, which can modulate the activity of complement receptors at the RPE, are locally produced in the retina [26,41] and by ARPE-19 cells (Figure 3; Supplementary Materials, Figure S2B–I). The mRNA expression and cellular protein levels of the stabilizing complement regulator, properdin, were increased after 24 h of H_2_O_2_ treatment (Figure 3A,C–E), but properdin secretion was not detected (Figure 3B, Supplementary Materials, Figure S4G). This indicated properdin storage in the stressed ARPE-19 cells (Figure 3C–E).

The transcription levels of additional complement components (*c3*, *c4a*, *c4b*, *cfb*, *cfd*, and *c5*) and soluble (*cfh*, *cfi*) and membrane-bound complement regulators (*cd46*, *cd59*) did not significantly change under oxidative stress conditions (Figure 3F,K; Supplementary Materials, Figure S2B–I).

However, we observed a change in cellular accumulation and the modulated secretion of complement components in the protein level through oxidative stress (Figure 3H–J,L–O). Central complement component *c3* was not regulated in mRNA and the protein secretion level by oxidative stress (Figure 3F,G; Supplementary Materials, Figure S4H), but we detected an increase in cellular C3 in immunostainings of ARPE-19 cells (Figure 3H–J). The secretion of C3 was more observable in younger compared to older ARPE-19 cells treated with H_2_O_2_ (Supplementary Materials, Figure S4B). A similar effect of cellular complement component accumulation and associated reduced secretion was detectable for complement regulator CFH (Figure 3L–O; Supplementary Materials, Figure S4I). However, *cfh* mRNA expression was not changed under oxidative stress (Figure 3K).

### 3.4. Autocrine Complement Receptor Activation Following Oxidative Stress Was Correlated with the Release of Proinflammatory and Proangiogenic Factors

**Figure 4:**
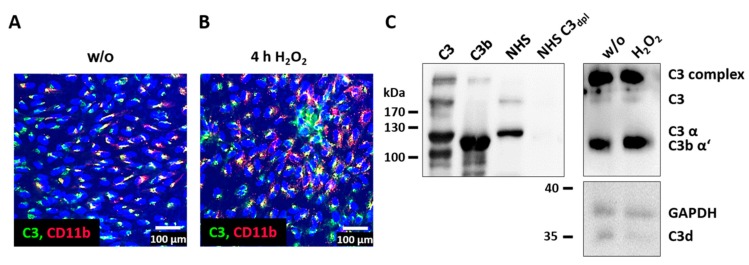
C3 and complement receptor CD11b were colocalized in ARPE-19. (**A**) Unstressed (w/o) and (**B**) H_2_O_2_-treated ARPE-19 cells were stained with anti-C3 (green) and anti-CD11b (red) antibodies. Overlapping staining signals (yellow) suggested a colocalization of C3 and CD11b. (**C**) C3 and activation products (C3b α’ and C3d) were detected in untreated and H_2_O_2_-treated ARPE-19 cells using a Western blot under reducing conditions (controls: native C3, C3b, human serum (NHS), and C3-depleted human serum (NHS C3_dpl_)). Full immunoblots are shown in the Supplementary Materials, Figure S3C,D; immunoblots were repeated twice.

Intracellular complement proteins and cellular complement receptors have previously been associated with the autocrine regulation of cell differentiation and cell physiology in T-cells as well as lung epithelial cells [20,42]. In line with this, we found a colocalization of CD11b and C3 in ARPE-19 cells (Figure 4A,B) and activated C3 fragments (C3b α’, C3d) in the ARPE-19 cells (Figure 4C), without adding any exogenous complement source.

**Figure 5:**
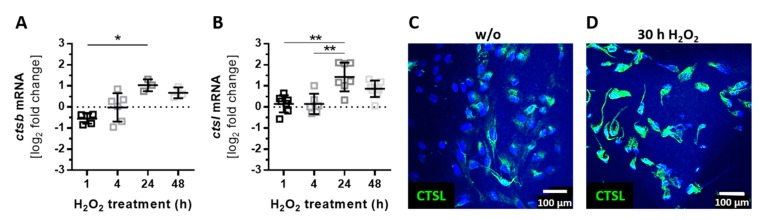
The expression of intracellular proteases was increased by oxidative stress in ARPE-19. (**A**) *Ctsb* and (**B**) c*tsl* mRNA expression increased 24 h after H_2_O_2_ treatment. This effect was confirmed on the protein level in immunostainings using an (**C**,**D**) anti-CTSL (green) antibody. (**A**,**B**) Mean with standard deviation is shown, * *p* ≤ 0.05, ** *p* ≤ 0.01, dotted line depicts untreated control; (**C**) w/o untreated control.

The intracellular cleavage of complement proteins into active fragments (independently of the systemic complement cascade) can be mediated by intracellular proteases such as cathepsin B (CTSB) and cathepsin L (CTSL) [17,18]. Both proteases were expressed by ARPE-19 cells, and they were upregulated following oxidative stress (Figure 5). The mRNA expression of *ctsb* and *ctsl* was increased after 24 h of H_2_O_2_ treatment (Figure 5A,B). We confirmed a higher concentration of CTSL in ARPE-19 cells under stress conditions also on the protein level (Figure 5C,D).

**Figure 6:**
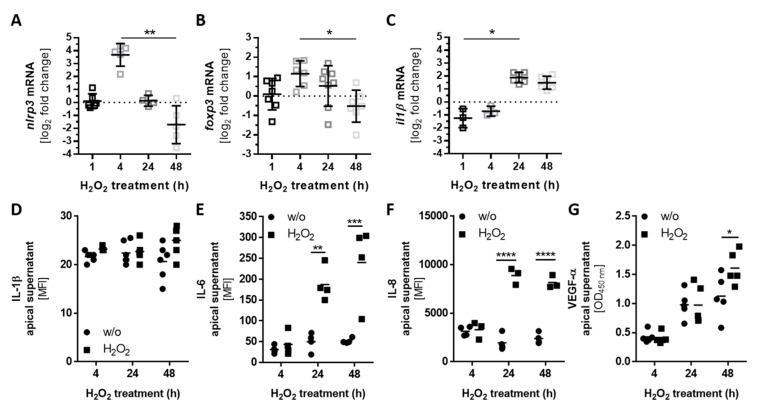
Increased *nlrp3* and *foxp3* mRNA expression correlated with proinflammatory and proangiogenic factor secretion. (**A**) *Nlrp3*, (**B**) *foxp3*, and (**C**) *il1β* mRNA levels increased either (**A**,**B**) 4 h or (C) 24 h and 48 h following H_2_O_2_ treatment. The proinflammatory cytokine release of (**D**) Interleukin (IL)-1β and (**E**) IL-6 was detected in stressed ARPE-19 cells. This was correlated with an enhanced secretion of the proangiogenic factors (**F**) IL-8 and (**G**) vascular endothelial growth factor (VEGF)-α in H_2_O_2_-treated cells. MFI: mean fluorescence intensity. Mean with standard deviation is shown, * *p* ≤ 0.05, ** *p* ≤ 0.01, *** *p* ≤ 0.001, **** *p* ≤ 0.0001; (**A**,**B**,**C**) dotted line depicts untreated control; (**D**–**G**) w/o untreated control; protein concentrations in the basal supernatants are shown in the Supplementary Materials, Figure S4J–L.

The activation of complement receptor signaling regulates the pro- and anti-inflammatory response in T- and RPE cells [24,43]. This can induce inflammasome activation and regulate the mammalian target of rapamycin (mTOR)-pathway, involving the FOXP3 transcription factor [24,25,44]. After the detection of H_2_O_2_-dependent regulation of complement receptors (Figure 2), cellular complement protein accumulation (Figure 3), cell-derived C3 colocalized with CD11b, and C3 activation products C3b and C3d in ARPE-19 cells (Figure 4), we hypothesized that the NLRP3 inflammasome and FOXP3 also play an autocrine, complement-dependent role in ARPE-19 cells treated with H_2_O_2_. This regulation would be independent of blood-derived complement components and would involve the release of cytokines and growth factors in stressed ARPE-19 cells (Figure 6).

Indeed, we detected an increased expression of *nlrp3* and *foxp3* mRNA after 4 h of H_2_O_2_ treatment (Figure 6A,B). Subsequent enhanced expression of *il1β* mRNA after 24 h and 48 h was associated with increased *nlrp3* levels in stressed ARPE-19 cells (Figure 6C). However, the mRNA expression of *il18* was not changed (Supplementary Materials, Figure S2J). Further, we found higher proinflammatory cytokine levels in the H_2_O_2_-treated ARPE-19 cell supernatants compared to the untreated controls (Figure 6D,E). IL-1β was slightly increased after treatment, while IL-6 was significantly elevated in the supernatant of stressed ARPE-19 cells.

Increased *foxp3* expression is an attribute of anti-inflammatory regulatory T-cells, which secrete mainly transforming growth factor (TGF)-β and IL-10. We did not detect a change in *tgf-β* expression (Supplementary Materials, Figure S2K) or IL-10 secretion in H_2_O_2-_treated ARPE-19 cells. Therefore, we assumed a proangiogenic function of *foxp3* in the cells, as previously reported [22,23]. In line with this, we observed an increase in IL-8 and VEGF-α concentration in the apical supernatant of stressed ARPE-19 cells (Figure 6F,G). This correlation between complement components, *foxp3* expression, and proangiogenic reactions in RPE cells needs to be further investigated.

As a side note, IL-17, interferon (IFN)-γ, IL-18, IL-2, and tumor necrosis factor (TNF)-α were not detected in the apical or basal supernatant of 4-, 24-, and 48-h untreated and H_2_O_2-_treated ARPE-19 cells (data not shown).

### 3.5. Olaparib Boosted the Proinflammatory Response of ARPE-19 Cells to Oxidative Stimuli

**Figure 7:**
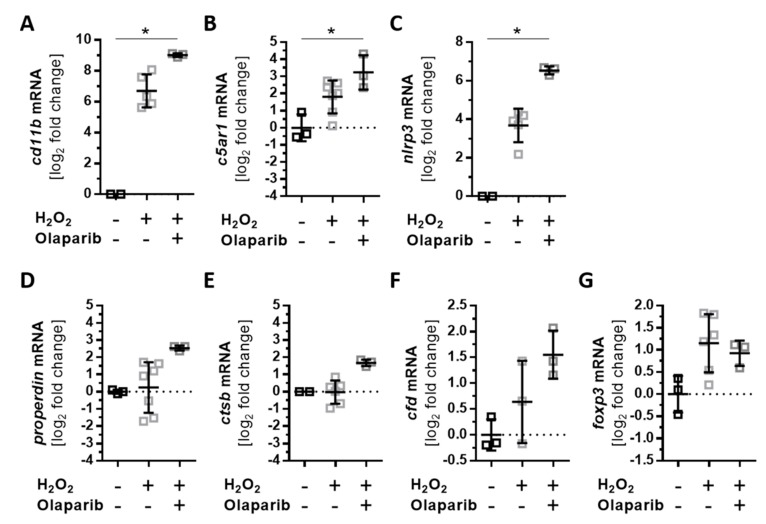
Olaparib enhanced oxidative stress-dependent expression changes in ARPE-19 cells**.** ARPE-19 cells were treated for 4 h with H_2_O_2_, and the effect of simultaneously added olaparib on transcription was investigated. (**A**) *Cd11b*, (**B**) *c5aR1*, and (**C**) *nlrp3* transcripts were significantly increased in olaparib-treated, stressed cells compared to unstressed cells. Olaparib also increased the expression of (**D**) *properdin*, (**E**) *ctsb*, and (**F**) *cfd*. (**G**) *Foxp3* mRNA levels were not changed in stressed ARPE-19 cells following olaparib addition. Mean with standard deviation is shown, * *p* ≤ 0.05; dotted line depicts untreated control.

Oxidative stress-induced cellular reactions have been previously ameliorated by an approved anticancer drug, olaparib, which is an inhibitor of poly(ADP-ribose) polymerase (PARP) [45,46,47]. We investigated the effects of olaparib on H_2_O_2_-dependent mRNA expression changes of complement receptors, components, and inflammation-related transcripts (Figure 7, Supplementary Materials, Figure S5). Oxidative stress increased the expression of *cd11b*, *c5ar1*, and *nlrp3* after 4 h of H_2_O_2_ treatment. This was further enhanced by olaparib treatment (Figure 7A–C). An increase in *properdin* and *ctsb* transcripts was observed after 24 h following oxidative stress alone (Figure 3A and Figure 5A). A combination of H_2_O_2_ and olaparib accelerated this reaction, with a significant increase in *properdin* and *ctsb* mRNA expression after only 4 h (Figure 7D,E). The expression of *cfd* (Supplementary Materials, Figure S2E) was not altered under oxidative stress; however, H_2_O_2_ and olaparib together increased *cfd* transcript levels (Figure 7F). Olaparib did not interfere with the transcription of *foxp3* (Figure 7G) and other transcripts (*c3*, *c4a*, *c5*, *cfb*, *cfh*, *cfi*, *c3ar*, and *ctsl*) (Supplementary Materials, Figure S5) in ARPE-19 cells treated with H_2_O_2_.

## 4. Discussion

The RPE is exposed to high-energy light, and it conducts the phagocytosis of oxidized photoreceptor outer segments. Both of these processes are accompanied by a rapid release of reactive oxygen species [6,48,49]. Reactive oxygen species, including H_2_O_2_, are on the one hand major cellular stressors [6,50] and on the other hand cellular survival factors [3,51]. Antioxidants are decreased in light-exposed retinae, allowing the intraocular accumulation of H_2_O_2_ [52]. We used H_2_O_2_ treatment to mimic physiological oxidative stress in serum-free cultivated ARPE-19 cells to investigate the endogenous complement response in ARPE-19 cells independent of external complement sources [53,54].

Oxidative stress increased the concentration of the complement regulators CFH and properdin and the central complement protein C3 in ARPE-19 cells in a time-dependent manner, without access to any extracellular complement source. Previous studies have mostly reported a reduced expression of *cfh* mRNA in RPE cells exposed to oxidative stress [28,29,30,31], but these studies did not include further CFH protein analysis. Our reported CFH protein accumulation after H_2_O_2_ treatment in polarized, monolayer ARPE-19 cells (using immunohistochemistry) is in contrast to reduced CFH protein detection results in Western blots of non-in vivo-like cultivated ARPE-19 cells following H_2_O_2_ treatment [30].

However, it is known that intracellular CFH can enhance the cleavage of endogenously expressed C3 through a cathepsin L (CTSL)-mediated mechanism [55]. The concentrations of lysosomal protease CTSL and the central complement protein C3 were both enhanced under oxidative stress conditions in ARPE-19 cells. Previous studies of RPE cell-derived complement components only focused on *c3* mRNA expression, which was not changed under low H_2_O_2_ concentrations [56]. We went a step further and showed that C3 was accumulated in the ARPE-19 cells following oxidative stress. This ARPE-19 cell-dependent local accumulation of C3 was also shown for ARPE-19 cells treated with cigarette smoke [27].

If C3 is activated in the blood, CFH serves as a negative regulator and properdin as a positive regulator. We showed for the first time that oxidative stress increased *properdin* mRNA expression in ARPE-19 cells. This resulted in a higher properdin protein concentration in these cells, which may promote cellular C3 cleavage. In summary, our data described a local production of complement proteins in ARPE-19 cells and an enhanced cellular storage of complement proteins in the cells after H_2_O_2_ treatment. This cellular accumulation suggests an autocrine, cellular function of complement proteins in ARPE-19 cells following oxidative stress that is independent of external complement protein sources.

Our studies revealed a colocalization of accumulated, endogenous C3 with complement receptor 3 (CR3, subunit CD11b) in ARPE-19 cells exposed to oxidative stress and an increase in CD11b after 4 h. CR3 expression has been associated with inflammasome activation as a reaction to complement components and/or oxidative stress in white blood cells and RPE cells [57,58]. In agreement with this association, the addition of H_2_O_2_ to ARPE-19 cells increased the time-dependent expression of *nlrp3* and *il-1β* mRNA and subsequently enhanced the secretion of proinflammatory cytokines.

Inflammasome activation can be triggered by reactive oxygen species and has been associated with lipid peroxidation end products and phototoxicity in RPE cells [59,60]. The involvement of the complement components in this oxidative stress response of RPE cells has only been described in relation to extracellularly added anaphylatoxins so far [24], but an endogenous complement of RPE cells has not been suggested as a potential priming factor for the inflammasome. We detected activated C3 cleavage products in ARPE-19 cells, and previous studies have shown that activated C3a can be intracellularly generated in RPE cells independent of the systemic canonical complement system [32,33,34,35]. Further, C3 receptors were expressed (CR3, C3aR) and regulated (CR3) under oxidative stress in ARPE-19 cells, indicating a role for endogenous complement components in stressed ARPE-19 cells.

Cellular C3 is cleaved by lysosomal CTSL [18,55], and NLRP3 inflammasome activation depends on this CTSL activity [60]. It has been reported that CTSL inhibition reduces inflammasome activity in ARPE-19 cells exposed to oxidative stress [61]. These findings show the interaction between cell-specific complement component cleavage and inflammasome activity. It is already known that endogenous C3-driven complement activation is required for IL-1β and IL-6 secretion, as well as for inflammasome activation in immune cells [62]. Our data suggest that proinflammatory cytokine secretion may also be an autocrine mechanism in ARPE-19 cells associated with complement components and receptors.

In addition to C3, C5 has been identified as a key player in cell homeostasis [24]. The *c5aR1* receptor is expressed in RPE cells [63,64] and was increased after oxidative stress treatment. *C5* mRNA expression was not changed, and the biologically highly active C5a fragment, a ligand for C5aR1 with a very low biological half-life (approximately 1 min [65,66]), was not detected in our study. The rapid C5a–C5aR interaction might have interfered with our detection schedule. However, C5aR1 stimulation is associated with IL-8 and VEGF-α secretion in ARPE-19 cells [63,64]. The increased secretion of these proangiogenic factors was also observed following the H_2_O_2_ stimuli. The signaling pathway is not exactly known so far, but exclusive C5aR1 activation by non-ARPE-19 cell components can be excluded. In regulatory T-cells, the transcription factor FOXP3 promotes the expression of IL-8 [22], and in bladder cancer cells, a knock-down of *foxp3* has resulted in the reduced expression of *vegf-α* [23]. *Foxp3* mRNA was expressed in ARPE-19 cells and increased under oxidative stress conditions. Previous studies have shown that extracellular C5a can activate FOXP3 in ARPE-19 cells, which is associated with increased IL-8 secretion [25]. We showed that this could also be due to the endogenous activation of C5aR1 following oxidative stress in RPE cells.

These changes in expression and cellular complement protein accumulation following oxidative stress were time-dependent (Supplementary Materials, Figure S6). The first changes in complement receptor (CD11b, C5aR1) and component (CFH, C3) levels in the ARPE-19 cells occurred after 4 h and were accompanied by changes in *nlrp3* and *foxp3* mRNA expression. Downstream alterations in properdin expression, intracellular proteases, and an increase in the epithelial–mesenchymal transition marker as well as a loss of tight junctions were described. This indicates that complement receptor signaling may be involved in the early response of ARPE-19 cells to H_2_O_2_ treatment.

Oxidative stress-related cell damage in ARPE-19 cells and retinal degeneration in mouse models of RPE degeneration, as well as hereditary retinal degeneration, were successfully ameliorated using olaparib in previous studies [45,46,47]. Olaparib is a clinically developed poly(ADP-ribose) polymerase inhibitor that was developed for cancer treatment by blocking the DNA repair mechanism. ARPE-19 cells were resistant to H_2_O_2_-induced mitochondrial dysfunction and to energy failure when olaparib was added [45]. We asked the following question: can olaparib also normalize complement-associated proinflammatory expression profiles in H_2_O_2_-treated cells? Surprisingly, olaparib accelerated the effect of oxidative stress in ARPE-19 cells and enhanced the expression of complement receptors, complement components, and *nlrp3* mRNA. This shows that the endogenous complement-related, proinflammatory response of ARPE-19 cells could be correlated with defective DNA repair mechanisms.

Finally, it needs to be pointed out that this analysis of endogenous complement components and oxidative stress reaction was primarily set up to generate the first data describing an RPE cell-dependent complement reaction. We took advantage of the most commonly used in vitro RPE model (the ARPE-19 cell line), which expresses well-characterized RPE-specific markers [67,68] and provides an unlimited genetic and environmentally identical availability without any risk of contamination with other or undifferentiated cell types. However, it needs to be considered that this model system bears a higher risk of undergoing an epithelial–mesenchymal transition because of long-term cultivation, showing limitations in measuring transepithelial resistance and less expressed RPE-specific markers compared to primary or stem-cell-derived RPE cells [68]. To follow up with this intriguing line of thinking, future studies are needed to verify these cell-associated complement functions in primary or stem-cell-derived RPE cells with different genetic and environmental backgrounds.

## 5. Conclusions

Oxidative stress and activation of the complement system cause retinal degeneration, but the mechanism behind this is still a matter of investigation. We showed for the first time that oxidative stress can increase endogenous ARPE-19 cell complement components and receptors and that the process was associated with the release of proinflammatory and proangiogenic factors.

Our data offer a steppingstone for numerous further investigations regarding the function of a cell-associated complement system in primary human RPE. Many questions were raised during this project: How are the complement components activated? Independent of external complement sources, what is (are) the signaling pathway(s) of the complement receptors? How are inflammasome regulation and FOXP3 activity modulated by endogenous complement components in RPE cells? Can endogenous complement factors be targeted to affect cell-associated signaling pathways? These new perspectives will hopefully help to decipher the function of intracellular complement components in retinal health and disease and offer new strategies for the treatment of retinal degeneration.

## Supplementary Materials

The following are available online at https://www.mdpi.com/2076-3921/8/11/548/s1, Figure S1: ARPE-19 cells showed a stable, confluent monolayer, and H_2_O_2_ treatment increased the expression of epithelial–mesenchymal transition markers; Figure S2: H_2_O_2_ treatment did not influence the transcription levels of several genes in ARPE-19 cells; Figure S3: The secretome of ARPE-19 cells was influenced by cell passages and H_2_O_2_ addition; Figure S4: The stable expression of complement components and related genes after Olaparib and oxidative stress treatment in ARPE-19 cells; Figure S5: Full immunoblots for Figure 2H and Figure 4C; Figure S6: Time-dependent changes of H_2_O_2_ treatment in ARPE-19 cells; Table S1: Primary and secondary antibodies; Table S2: QuantiTec PrimerAssays; Table S3: In-house-designed RT-qPCR primers.
