# Supplementary material for: Oxidative Stress Increases Endogenous Complement-Dependent Inflammatory and Angiogenic Responses in Retinal Pigment Epithelial Cells Independently of Exogenous Complement Sources
Source: Antioxidants (Basel). 2019 Nov 13;8(11):548. doi: 10.3390/antiox8110548 (PMC6928869; doi:10.3390/antiox8110548)
Supplement: Supplementary file 1 [file antioxidants-08-00548-s001.pdf]

Supplementary Data

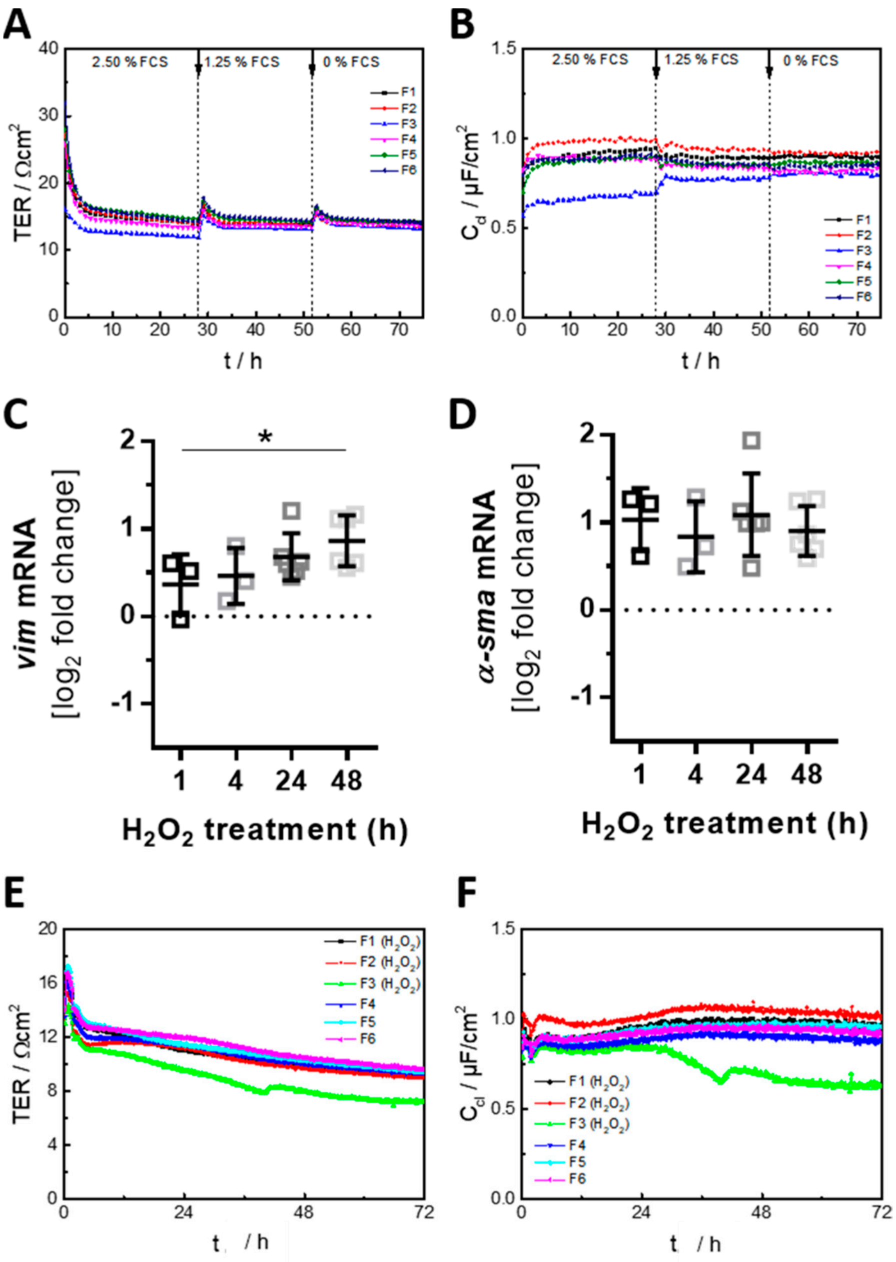

Figure 1. ARPE-19 cells showed a stable, confluent monolayer and H_2O_2 treatment increased expression of epithelial-mesenchymal transition markers. ARPE-19 cells were cultivated under *in vivo*-like conditions and pre-treatment reduction of FCS concentrations did not interfere with **(A)** TER or **(B)** capacitance of the cells. Following FCS reduction, the cells were treated for either 1, 4, 24 or 48 h with H_2O_2. **(C)** *vim* and **(D)** *α-sma* transcription was increased compared to the untreated control. Mean with standard deviation is shown, * $p \leq 0.05$, dotted line depicts untreated control. **(E)** TER and **(F)** capacitance were not altered by H_2O_2 addition.

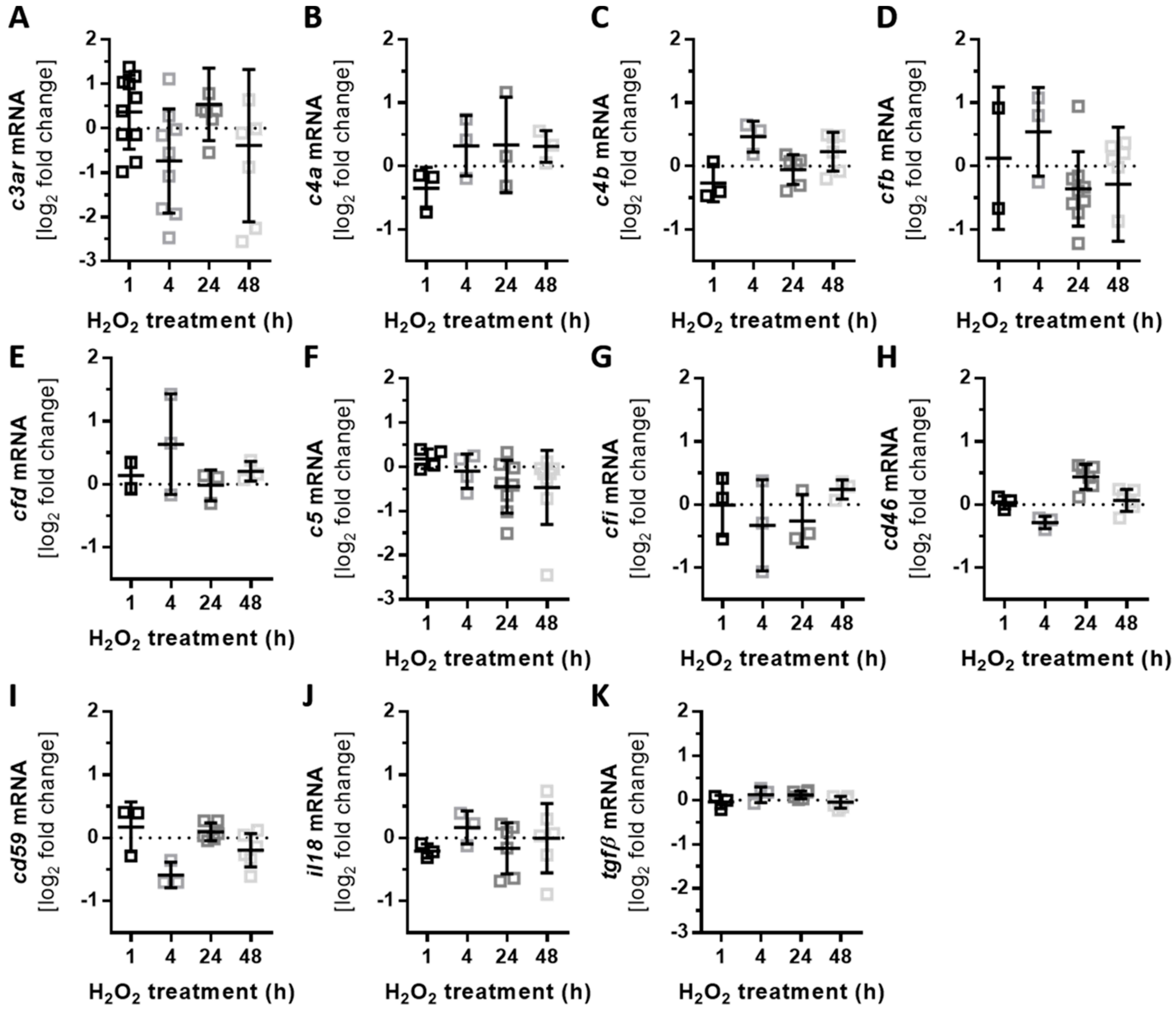

Figure 2. H_2O_2 treatment did not influence the transcription levels of several genes in ARPE-19 cells. ARPE-19 cells were treated for either 1, 4, 24 or 48 h with H_2O_2. mRNA levels were not significantly changed for: **(A)** *c3ar*, **(B)** *c4a*, **(C)** *c4b*, **(D)** *cfb*, **(E)** *cfd*, **(F)** *c5*, **(G)** *cfi*, **(H)** *cd46*, **(I)** *cd59*, **(J)** *il18* and **(K)** *tgfβ*. Mean with standard deviation is shown, dotted line depicts untreated control.

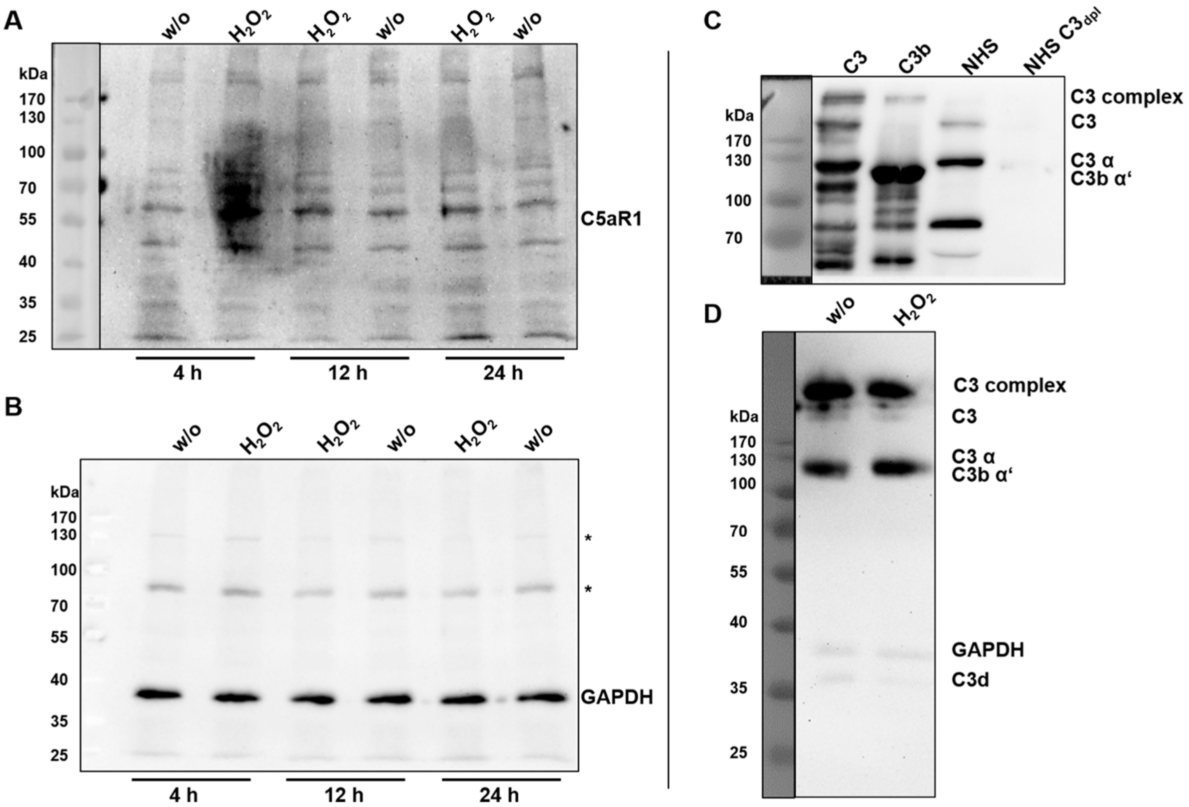

Figure 3. Full immunoblots for Figures 2H and 4C. Blots were sequentially developed: **(A, B)** 1st antibody anti-C5aR, **(B)** 2nd antibody (not determined), 3rd antibody anti-GAPDH. * Signals developed after 2nd antibody (not determined) development. **(C, D)** show full blots for anti-C3 antibody.

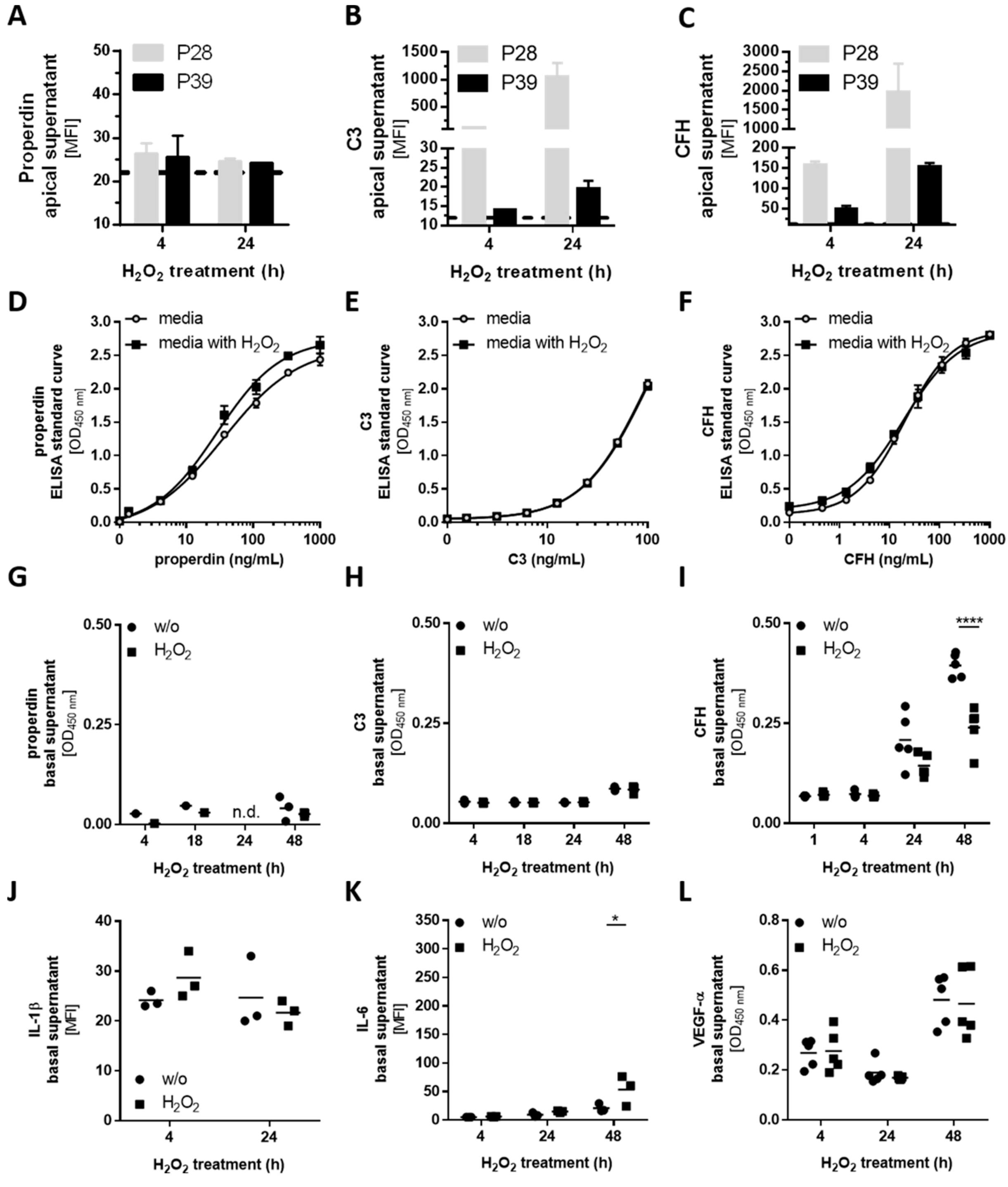

Figure 4. Secretome of ARPE-19 cells was influenced by cell passages and H_2O_2 addition. **(A - B)** ARPE-19 cells with passages 28 or 39 (latter used in the rest of this study) were treated for either 4 or 24 h with H_2O_2. The protein concentration of **(A)** properdin, **(B)** C3 and **(C)** CFH was determined in the apical supernatant using a multiplex complement immunoassay: **(A)** Properdin was determined at very low concentrations in the cell supernatants using this assay. **(B)** C3 and **(C)** CFH showed higher levels in cell supernatants with lower passage number (P28) than in supernatants of ARPE-19 cells with a higher passage number (P39). **(D - F)** Added H_2O_2 did not significantly alter the standard curves of **(D)** properdin, **(E)** C3 or **(F)** CFH in the used ELISA for secretome analysis (used for Figures 3B, G, L and S3G, H, I). **(G – L)** Concentrations of complement proteins and cytokines in basal supernatants of non-treated and H_2O_2-treated ARPE-19 cells were different compared to apical supernatants (compare with Figures 3B, G, L and Figures 6D, E, G): **(I)** CFH and **(K)** IL-6 showed a significant difference in non-treated versus treated basal supernatants. Basal protein levels of **(G)**

Properdin, **(H)** C3**, (J)** IL1-β or **(L)** VEGF-α were either **(G, H, J)** very low or did not show significant differences for non-treated versus treated samples.

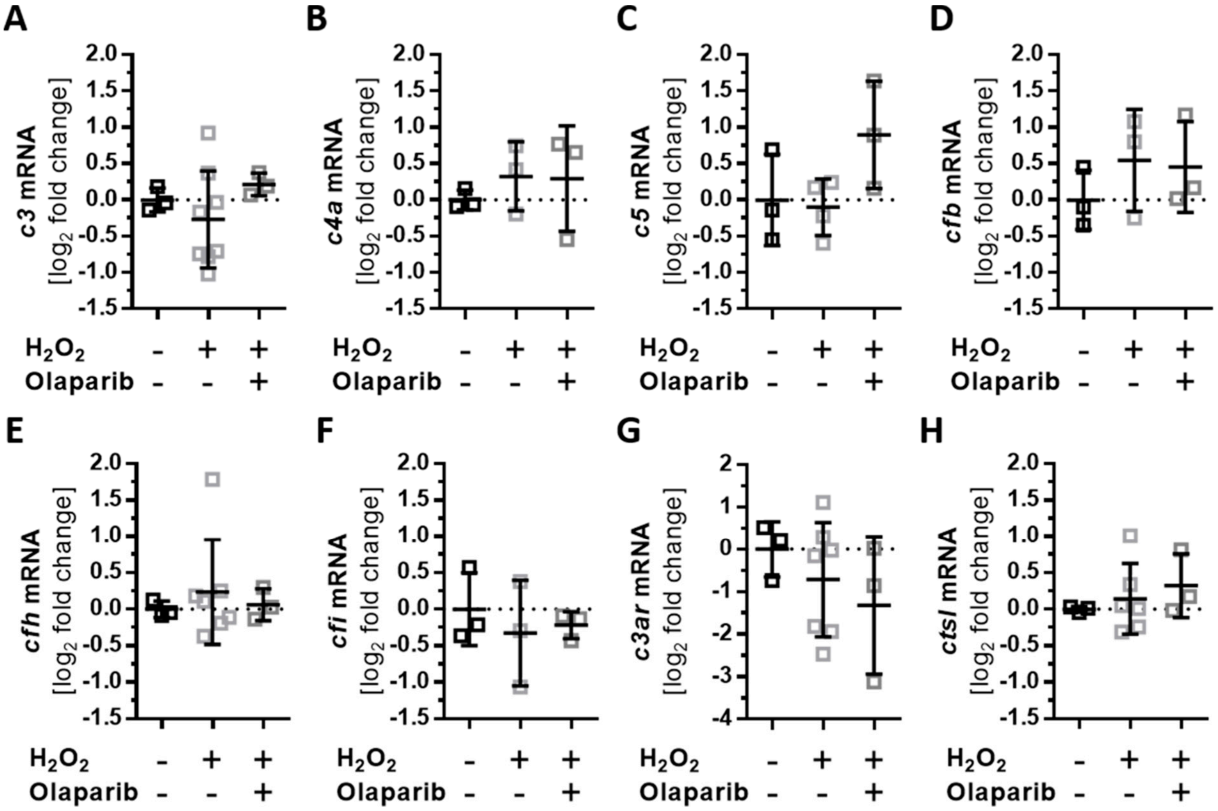

Figure 5. Stable expression of complement components and related genes after Olaparib and oxidative stress treatment in ARPE-19 cells. ARPE-19 cells were treated for 4 h with H_2O_2 and the effect of simultaneously added olaparib on transcription was investigated. (A) *c3,* (B) *c4a*, (C) *c5*, (D) *cfb*, (E) *cfh*, (F) *cfi*, (G) *c3aR* and (H) *ctsl* did not significantly change in stressed ARPE-19 cells following olaparib addition.

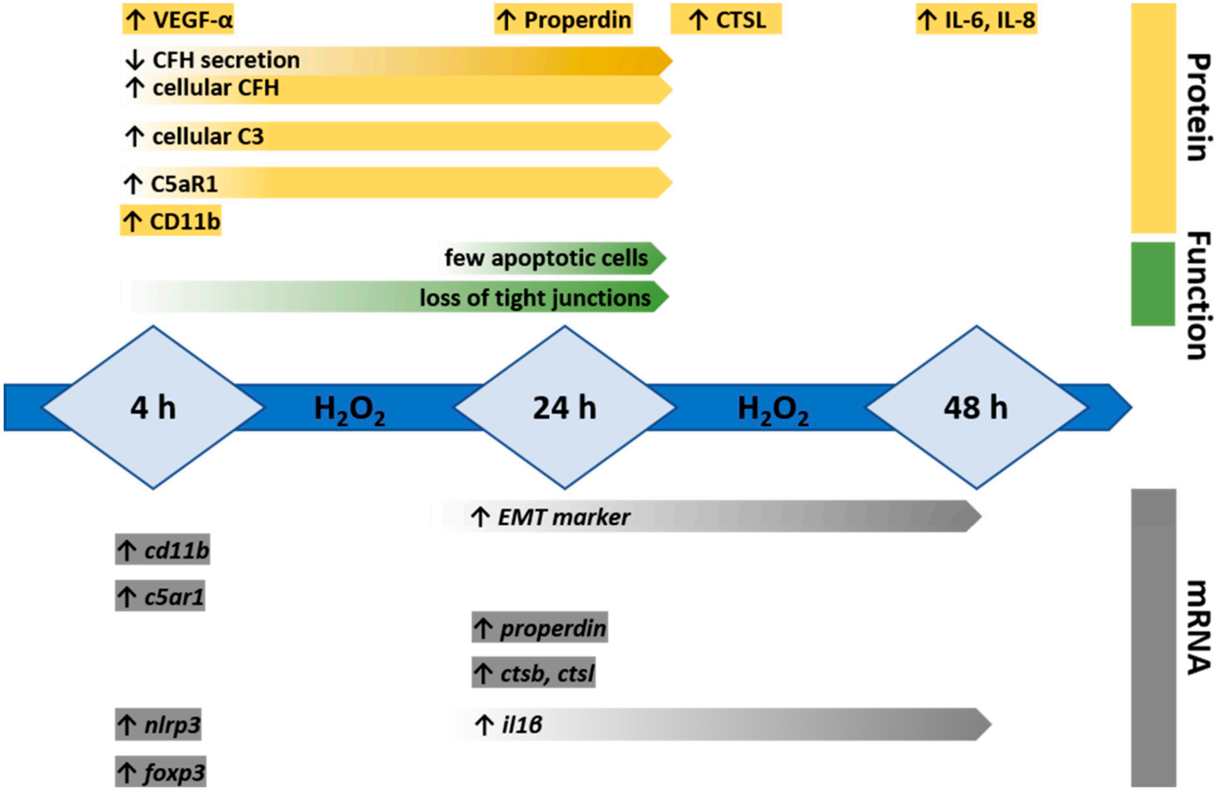

Figure 6. Time-dependent changes of H_2O_2 treatment in ARPE-19 cells. ARPE-19 cells were treated for 4, 24 and 48 h with H_2O_2. Changes in mRNA expression (grey), function (green) and on protein level (yellow), which are described in this manuscript, are summarized in this scheme.

Sup. Table S1: Primary and secondary antibodies.

Primary antibody	Species	Company	Catalogue number	Dilution / Concentration
anti-ZO-1	rabbit	ThermoFisher	61-7300	IS 1: 300
anti-CD11b	goat	Biorbyt	orb19554	IS 1:500
anti-C5aR1	mouse	Hycult	HM2094	IS 1:100, WB 1:1000
anti-GAPDH-HRP	rabbit	Cell signaling technology	3683	
anti-Properdin	goat	Complement Technology	A239	IS 1: 250
anti-C3	goat	Bio Rad	AHP1752	IS 1:250
anti-C3	rabbit	Abcam	Ab181147	WB 1: 1000
anti-CFH	goat	Quidel	A312	IS 1:250
anti-CFH	mouse	BioRad	MCA509G (clone Ox-249)	ELISA 1 μg/mL
anti-CFH	goat	Merck	341276	ELISA 1:5000
anti-CTSL	mouse	Abcam	ab6314	IS 1:500
anti-Properdin 1340	mouse	In house[1]	1340	ELISA 2 μg/mL
anti-Properdin mAb1	mouse	Quidel	A233	ELISA 1 μg/mL
**Secondary antibody**				
anti-mouse Ig-HRP	goat	Dianova	115-035-003	WB 1:5000
anti-rabbit Ig-HRP	goat	Dianova	111-035-003	WB 1:5000
anti-goat Ig-HRP	rabbit	Dianova	305-035-003	WB 1:5000 ELISA 1:10000
anti-goat IgG Cy3	donkey	Dianova	705-165-147	IS 1:500
anti-Mouse IgG (H+L) Alexa Fluor 488	donkey	ThermoFisher	AB_2534069	IS 1:500

WB – Western blot, IS – Immunostaining.

[1] Pauly D, Nagel BM, Reinders J, Killian T, Wulf M, Ackermann S, et al. A novel antibody against human properdin inhibits the alternative complement system and specifically detects properdin from blood samples. PLoS One. 2014;9(5):e96371.

Sup. Table S2: QuantiTec PrimerAssays.

mRNA transcript	name	Catalogue number
gapdh	Hs_GAPDH_1_SG	QT00079247
c3	Hs_C3_1_SG	QT00089698
c3ar	Hs_C3AR1_1_SG	QT00090398
cd11b	Hs_ITGAM_1_SG	QT00031500
c4a	Hs_C4A_1_SG	QT00237160
c4b	Hs_C4B_1_SG	QT00237167
c5	Hs_C5_1_SG	QT00088011
c5ar1	Hs_C5R1_1_SG	QT00997766
cd46	Hs_MCP_1_SG	QT00073689
cd59	Hs_CD59_1_SG	QT00035952
cathepsin b	Hs_CTSB_1_SG	QT00088641
cathepsin l	Hs_CTSL_1_SG	QT01664978
complement factor b	Hs_BF_1_SG	QT00012138
complement factor d	Hs_CFD_1_SG	QT00212191
complement Factor h	Hs_CFH_1_SG	QT00001624
complement Factor i	Hs_CFI_1_SG	QT00213794
complement Factor p	Hs_CFP_1_SG	QT00010514
nlrp3	Hs_NLRP3_1_SG	QT00029771
forkhead-box-protein P3	Hs_FOXP3_1_SG	QT00048286

Sup. Table 3: In-house designed RT-qPCR primers.

mRNA transcript	sequence
il1ß	fw: CTCGCCAGTGAAATGATGGCT<br>rv: GTCGGAGATTCGTAGCTGGAT
il18	fw: ACTGTAGAGATAATGCACCCCG<br>rv: AGTTACAGCCATACCTCTAGGC
tgfß	fw: CATAGCTGACTTCAAGATGTGGT<br>rv: CCTAGTGAGACTTTGAACCGT
vim	fw: TGTCCAAATCGATGTGGATGTTTC<br>rv: TTGTACCATTCTTCTGCCTCCTG
α-sma	fw: GCCTTGGTGTGTGACAATGG<br>rv: AAAACAGCCCTGGGAGCAT

fw - forward primer, rv - reverse primer.
